# Wanted, a Climate for Bronchitis

**Published:** 1899-07-01

**Authors:** 


### WANTED, A CLIMATE FOR BRONCHITIS.

"Sincerity" writes: May I venture to ask through your columns what place, district, or climate in this country is most suitable for patients suffering from chronic bronchitis and asthma, who are ill every year from the climate of Yorkshire? Would Morecambe Bay be suitable?

